# Peripheral Acid Sphingomyelinase Activity Is Associated with Biomarkers and Phenotypes of Alcohol Use and Dependence in Patients and Healthy Controls

**DOI:** 10.3390/ijms19124028

**Published:** 2018-12-13

**Authors:** Christiane Mühle, Christian Weinland, Erich Gulbins, Bernd Lenz, Johannes Kornhuber

**Affiliations:** 1 Department of Psychiatry and Psychotherapy, Friedrich-Alexander University Erlangen-Nürnberg (FAU), D-91054 Erlangen, Germany; christian.weinland@uk-erlangen.de (C.W.); bernd.lenz@uk-erlangen.de (B.L.); johannes.kornhuber@uk-erlangen.de (J.K.); 2 Department of Molecular Biology, University of Duisburg-Essen, D-45259 Essen, Germany; erich.gulbins@uni-due.de; 3 Department of Surgery, University of Cincinnati, Cincinnati, OH 45267-0558, USA

**Keywords:** acid sphingomyelinase, alcohol dependence, liver enzymes, sphingolipid metabolism, withdrawal

## Abstract

By catalyzing the hydrolysis of sphingomyelin into ceramide, acid sphingomyelinase (ASM) changes the local composition of the plasma membrane with effects on receptor-mediated signaling. Altered enzyme activities have been noted in common human diseases, including alcohol dependence. However, the underlying mechanisms remain largely unresolved. Blood samples were collected from early-abstinent alcohol-dependent in-patients (*n*[♂] = 113, *n*[♀] = 87) and matched healthy controls (*n*[♂] = 133, *n*[♀] = 107), and analyzed for routine blood parameters and serum ASM activity. We confirmed increased secretory ASM activities in alcohol-dependent patients compared to healthy control subjects, which decreased slightly during detoxification. ASM activity correlated positively with blood alcohol concentration, withdrawal severity, biomarkers of alcohol dependence (liver enzyme activities of gamma-glutamyl transferase, alanine aminotransferase, aspartate aminotransferase; homocysteine, carbohydrate-deficient transferrin; mean corpuscular volume, and creatine kinase). ASM activity correlated negatively with leukocyte and thrombocyte counts. ASM and gamma-glutamyl transferase were also associated in healthy subjects. Most effects were similar for males and females with different strengths. We describe previously unreported associations between ASM activity and markers of liver damage and myelosuppression. Further research should investigate whether this relationship is causal, or whether these parameters are part of a common pathway in order to gain insights into underlying mechanisms and develop clinical applications.

Part of this work has been presented as a poster at the 1st International Symposium “Neurodevelopment and CNS vulnerability” in Erlangen, Germany in September 2018.

## 1. Introduction

Yearly, nearly 2.5 million deaths worldwide are attributable to alcohol use, in addition to other consequences of alcohol-related diseases and injuries (World Health Organization: Global status report on alcohol and health). Various risk factors and mechanisms have been suggested to play a role in the development and maintenance of alcohol-use disorders [1]. Genetic components account for 50%–60% according to twin, adoption, and family studies [2]. Prenatal hyperandrogenization [3,4] may partially be responsible for the two-fold higher prevalence of alcohol dependence in males compared to females. Disturbances in sphingolipid metabolism have been identified in human studies and animal models of psychiatric disorders [5], including major and mild depression [6,7,8], which share a high comorbidity with alcohol dependence.

Sphingolipids play an increasingly recognized role in neuronal function in the brain, not only by serving as a membrane component to form a physical barrier. They also influence the local composition of the plasma membrane, the localization and activity of proteins and, thus receptor-mediated signaling, in addition to their own actions as ligands [9]. Current research specifically focuses on enzymes crucial for maintaining the balance between the pro-apoptotic ceramide and its anti-apoptotic metabolite sphingosine 1-phosphate (the so-called “rheostat”) in the context of various physiological and pathophysiological conditions [10]. Sphingomyelinases and ceramidases are involved, for example, in ceramide-mediated signal transduction required for apoptosis, differentiation, and other cellular (including inflammatory) responses, in intracellular cholesterol trafficking and metabolism, as well as in lysosomal degradation of sphingomyelin and ceramide [11]. Two further pathways, de novo biosynthesis and the salvage pathway, lead to the generation of ceramide.

Acid sphingomyelinase (ASM, EC 3.1.4.12), encoded by the gene *SMPD1*, catalyzes the hydrolysis of the abundant membrane lipid sphingomyelin into ceramide and phosphorylcholine. Altered enzyme activities have been noted in a variety of common human diseases [12]. In alcohol dependence, levels of both the lysosomal [13] and secretory form (S-ASM) of the enzyme are increased, and decrease gradually during withdrawal treatment in male, as well as female, patients [14,15]. Consequently, plasma glycerophospholipid and sphingolipid species are also dysregulated in alcohol-dependent patients [16]. In ethanol-fed mice, tissue ASM activity is increased [17,18]. To our knowledge, there are no further published studies on the influence of the exposure of animals or cultured cells to ethanol on peripheral or culture supernatant S-ASM levels, except for one report. No alteration of serum S-ASM activity was detected in both transgenic mice overexpressing ASM and wildtype mice in a two-bottle free-choice drinking paradigm with a gender-balanced design [19]. Stimulation of the neutral sphingomyelinase by ethanol likewise contributes to alterations in the sphingomyelin/ceramide balance [20,21].

Hepatotoxicity is a major consequence of alcohol misuse. Of note, liver damage could also result from components of alcoholic beverages beyond their ethanol content. Both in experimental models of chronic ethanol-induced steatohepatitis, and patients with severe chronic alcohol-related liver disease, the immunoreactivity and ceramide content are increased [22]. Interestingly, an accumulation of ceramide and elevated levels of S-ASM have also been found in non-alcoholic fatty liver disease [23]. ASM knockout mice are resistant to alcohol-mediated fatty liver and cell death [24]. Inhibition of ASM by imipramine blocked the ethanol-induced ASM activation and ceramide generation, resulting in amelioration of hepatic steatosis in ethanol-fed mice [18]. Likewise, treatment of ethanol-fed rats with antioxidants, for example, *N*-acetylcysteine, reduced the severity of chronic alcohol-related steatohepatitis, possibly attributable to the observed decreased expression of inflammatory mediators, reduced acid sphingomyelinase activity, and lowered ceramide load [25]. The role of ASM likely involves sensitization of hepatocytes to the cytotoxic effects of TNFalpha [24] and regulation of autophagy [26].

The lack of reliable markers of alcohol consumption is a major obstacle to the diagnosis and treatment of alcohol dependence. Interviews and subjective questionnaires have their limitations, particularly because subjects are known to downplay the extent of their drinking behavior. Direct measurement of alcohol concentration in the breath, blood, or urine does not provide information more than a few hours beyond the most recent consumption of alcohol [27], or it requires special equipment with a high cost (ethyl glucuronide, ethyl sulfate, phosphatidylethanol) [28]. Currently available indirect biochemical markers, including carbohydrate-deficient transferrin (CDT, a form of the serum iron-carrying protein transferrin, with altered carbohydrate composition), mean corpuscular volume of erythrocytes (MCV), as well as the liver enzymes gamma-glutamyl transferase (GGT), alanine aminotransferase (ALT, also glutamic-pyruvic transaminase GPT), and aspartate aminotransferase (AST, also glutamic-oxaloacetic transaminase GOT), react to steady and significant alcohol intake over weeks or months, but suffer from relatively low sensitivity and specificity, and an uncertain time window of detection [28,29].

Furthermore, there is a need for reliable predictors of relapse after withdrawal treatment, which is a common problem in alcohol dependence, resulting in a rate of up to 85% in the absence of further support after the initial detoxification phase [30]. A number of known risk factors have been identified, albeit with limited accuracy and a high cost and time investment, which restricts their clinical applications for the identification of patients at risk of relapse and for individualized treatment [31].

In our large and sex-balanced cohort of alcohol-dependent patients and matched healthy controls, we aimed at characterizing the readily quantifiable activity of peripheral S-ASM with respect to the phenotype and known biomarkers of alcohol dependence, with particular emphasis on liver parameters. Moreover, we evaluated the diagnostic performance of this enzyme in discriminating between patients and controls, and predicting relapse as assessed as alcohol-related readmissions to the hospital.

## 2. Results

### 2.1. Elevated S-ASM Activity in Early-Abstinent Alcohol-Dependent Patients

**Figure 1:**
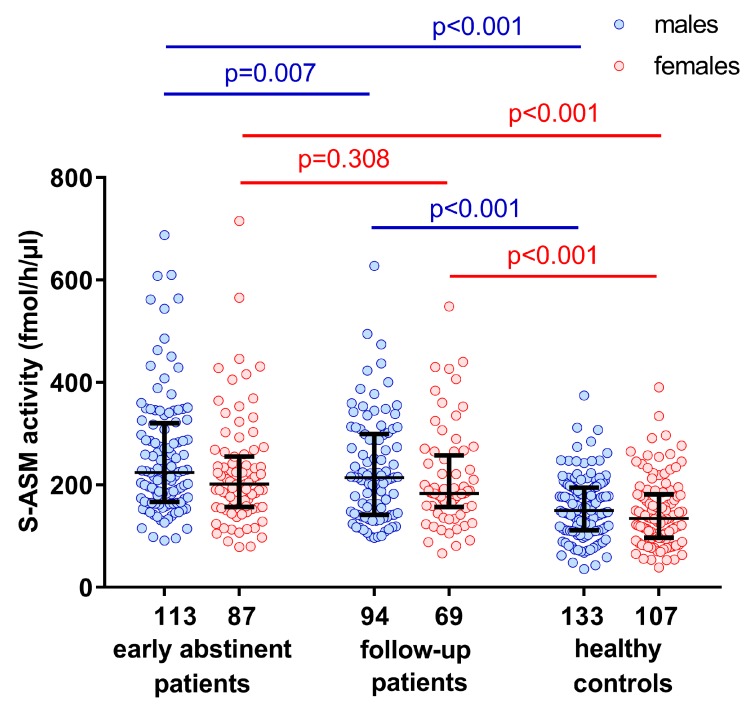
Sex-specific activity of the secretory acid sphingomyelinase (S-ASM) in alcohol-dependent male and female patients during early abstinence and follow-up, compared to healthy control subjects. Boxplots show individual data, and the median and interquartile range. The numbers of male and female individuals are provided below the x-axis. *p*-values were calculated using the Mann–Whitney *U* test except for the pair-wise comparison for patients where the Wilcoxon signed-rank test was applied.

We quantified the serum S-ASM activity in our cohort of 200 severely alcohol-dependent patients, and 240 control subjects matched for age and sex (Table 1). We replicated previous findings of significantly increased levels of S-ASM activity in early-abstinent alcohol-dependent patients [14,15] in this considerably larger cohort. At recruitment during early abstinence, male as well as female patients both presented with 1.5-fold higher serum activities, compared to healthy controls (*p* < 0.001, Figure 1). In both patients and controls, the enzyme activity was 11% higher in males than in females (*p* = 0.049 and *p* = 0.100, respectively). On average, levels decreased slightly during withdrawal treatment by 9.4% (*p* = 0.007) and 1.5% (*p* = 0.308) for male and female patients, respectively, to levels that were still significantly higher than those of control subjects (*p* < 0.001). However, a decrease was only observed in about half of the patients (63% of males and 52% of females) during the approximately 5-day interval.

### 2.2. S-ASM Activity Is Positively Associated with Alcohol Levels at Admission and Withdrawal Severity

**Figure 2:**
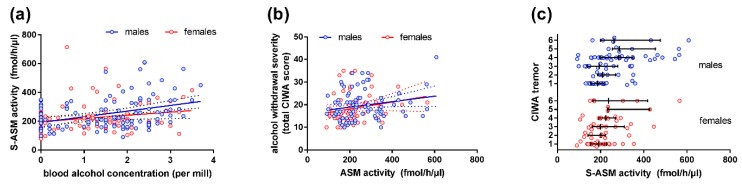
Correlation of serum S-ASM activity with blood alcohol levels and withdrawal in alcohol-dependent patients: (**a**) S-ASM activity is positively associated with blood alcohol levels of male and female patients at study inclusion; (**b**) S-ASM activity is positively associated with withdrawal severity in male and female patients assessed by the Clinical Institute Withdrawal Assessment for Alcohol revised scale (CIWA-Ar total score); (**c**) S-ASM activity is strongly positively associated with the CIWA-Ar sub-item tremor. S-ASM: secretory acid sphingomyelinase. Individual data with linear regression line and 95% confidence intervals (**a**,**b**) and boxplots with median and interquartile range (**c**).

Blood alcohol levels determined during recruitment varied from 0‰ to 3.7‰, and they correlated significantly with S-ASM activity (Rho = 0.315, *p* = 8.3 × 10^−6^, Figure 2a). This moderate effect was mainly driven by the male subgroup (Rho = 0.371, *p* = 7.7 × 10^−5^) and was much smaller in female patients (Rho = 0.214, *p* = 0.049). In previous experiments, we had ensured that, in our assay, the analysis of S-ASM activity was not confounded by the remaining alcohol concentrations in serum samples [14]. After subdividing the patients according to their predominantly consumed type of alcoholic beverage (beer *n* = 83, wine *n* = 43, hard liquor *n* = 25), no significant difference in alcohol concentrations at admission or in S-ASM activity was observed between these subgroups for all patients and for sex-specific analyses (all *p* > 0.05, Supplemental Figure S1).

To investigate the relationship between S-ASM activity and withdrawal severity, patients were asked at the follow-up visit to report their strongest withdrawal symptoms since study inclusion (CIWA-Ar scale). The cumulative score for ten sub-items (Supplemental Table S1) showed a significant correlation with S-ASM activity in the total cohort (Rho = 0.242, *p* = 0.003, Figure 2b). This correlation derived from the male patients (Rho = 0.194, *p* = 0.079), and, to a larger extent, from female patients (Rho = 0.267, *p* = 0.034). There was a particularly notable association between S-ASM and the sub-item scores for nausea/vomiting (Rho = 0.232, *p* = 0.005), and most strongly for tremor (Rho = 0.351, *p* = 1.4 × 10^−5^) in the total cohort with considerably stronger effects in the male patient subgroup (Figure 2c, Rho = 0.395, *p* = 2.2 × 10^−4^ in males vs. Rho = 0.250, *p* = 0.048 in females).

### 2.3. S-ASM Activity Is Strongly Associated with Liver Enzymes in Alcohol-Dependent Patients

**Figure 3:**
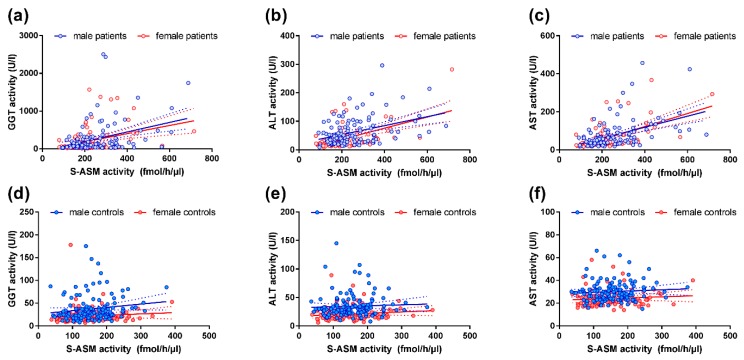
Correlation of serum S-ASM activity with liver enzymes in alcohol-dependent patients and healthy controls: (**a**–**c**) Associations for patients; (**d**–**f**) Associations for healthy controls. For control graphs, the strongest outliers deviating from the mean by more than two standard deviations were excluded from the graph (one for GGT, two each for ALT and AST each) to avoid distortion of the linear regression line and compression of the dataset. S-ASM: secretory acid sphingomyelinase, GGT: gamma-glutamyl transferase, ALT: alanine aminotransferase (glutamic-pyruvic transaminase, GPT), AST: aspartate aminotransferase (glutamic-oxaloacetic transaminase, GOT). Individual data with linear regression line and 95% confidence intervals.

The liver is severely damaged by excessive consumption of alcoholic beverages as indicated by an increase in activities of the liver enzymes GGT, ALT, and AST. In line with the elevated S-ASM activities in patients, we have found strong and highly significant correlations of S-ASM with these liver enzyme activities (Rho > 0.37 and *p* < 6 × 10^−8^ for all three enzymes, Table 2, Figure 3). Interestingly, the strength of these associations was similar for both male and female patients.

### 2.4. S-ASM Activity Is Associated with GGT and ALT Activity in Healthy Controls

Despite the much lower variations in GGT and ALT liver enzyme activities in healthy controls compared to patients, both were also positively (albeit weaker) correlated to the S-ASM activity (Table 2, Figure 3). The female subgroup contributed more to this effect. While alcohol blood levels or consumption were not assessed in the healthy control subjects and could be the mediators, S-ASM, however, was not associated with commonly utilized scales to detect alcohol misuse, using AUDIT (Rho = 0.085, *p* = 0.208) or CAGE scores (Rho = 0.038, *p* = 0.555). This was also true for sex-specific analyses. Of note, further exploratory analysis revealed an influence of age on S-ASM activity in this group of healthy individuals (Rho = 0.268, *p* = 2.6 × 10^−5^) which was clearly more relevant for males (Rho = 0.320, *p* = 1.8 × 10^−4^) than females (Rho = 0198, *p* = 0.041). Moreover, GGT activity was found to be age-dependent in this group (Rho = 0.286, *p* = 7.0 × 10^−6^), with a definitely larger effect from males (Rho = 0.352, *p* = 3.3 × 10^−5^) than females (Rho = 0.299, *p* = 0.002). In consideration of these two positive correlations, we cannot exclude that the observed association of S-ASM with GGT in healthy subjects is partially driven by an age effect. For ALT, a dependence on age was exclusively found in the female subgroup (Rho = 0.336, *p* = 4.0 × 10^−4^).

When we subdivided the group of healthy control subjects into those with at least one binge-drinking episode (≥5 standard drinks of ~13 g of alcohol per drink within 2 h) during the past 24 months (*n* = 36) versus those without a binge-drinking episode (n = 204), we unexpectedly observed slightly decreased S-ASM levels in binge-drinkers in comparison to the increased S-ASM levels in alcohol-dependent patients (Supplemental Table S2). However, considering the influence of age, these lower S-ASM activities might be due to the naturally lower age of the binge-drinking group, particularly the males.

Another unanticipated observation was that the association of S-ASM levels with liver enzymes strengthened in the healthy control sample after exclusion of binge-drinkers (GGT: Rho = 0.310, *p* = 6.3 × 10^−6^; ALT: Rho = 0.193, *p* = 0.006 for *n* = 204, compared to Table 2) whereas there was no statistical trend observed for the small binge-drinking group (*n* = 36, *p* > 0.3 for both enzymes). This was also true for the sex-specific analysis of GGT.

### 2.5. Comparison of S-ASM Activity with Additional Biomarkers of Alcohol Dependence

In addition to a clear elevation of the liver enzymes GGT, ALT, and AST, pathophysiological processes in alcohol-dependent patients lead to further alterations that can be detected in peripheral blood samples, including CDT, MCV (both often serving as biomarkers for alcohol dependence), and homocysteine. Of note, S-ASM activities were also associated with these parameters, but these relationships were weaker than those with liver enzymes, and they also showed different strengths for male and female subgroups (Table 2). For CDT, the association was strong and highly significant for the total group, as well as for males and females separately. For homocysteine, it was moderate and highly significant for the total group, but only for males separately. For MCV, the association was weak and only found for females.

We next analyzed the quality of S-ASM as a biomarker for alcohol dependence by comparing sensitivity and specificity with those of established parameters. The predictive power of S-ASM activity in our cohort, as judged from the area under the curve (AUC = 0.771), was in a similar range, but it was lower than that of classically used biomarkers CDT (0.868), GGT (0.853), and MCV (0.799) in our sample (Table 3). Due to its high correlation with these parameters, adding S-ASM to the biomarkers listed in Table 3, in a binary logistic regression model that included sex and age, did not clearly improve the prediction (correct classification of one more patient). This marker would, thus, probably not add clinically relevant information to the current practice to separate alcohol-dependent patients from non-alcohol-dependent subjects.

When comparing males and females, the differentiation capacity of the analyzed parameters, as indicated by the AUC, was similar, with a maximum of 5% difference in AUC. S-ASM and CDT differentiated slightly better for males, and GGT, MCV, ALT, AST, and Hcy for females. This was despite considerable variation in the cut-point determined by the Youden index. For the S-ASM activity, the optimal cut-point was nearly 50% higher (224 fmol/h/µL) in males than in females (151 fmol/h/µL), which might reflect the weak sex difference in patients and trend difference in controls (Table 1). The Youden cut-point for males was also markedly higher than for females for the liver enzyme activities GGT, ALT, and AST, with a factor between 1.3 and 2.1 (Table 3) related to the sex differences for these enzymes in patients and controls (Table 1).

We included creatine kinase (CK) in our analysis, which is typically assayed as a marker of muscle damage, because alcohol has been found to lead to a rapid increase in plasma CK activity in rats [33]. Moreover, raised levels of CK have been detected in various psychiatric conditions, including alcohol dependence [34]. In addition, CK differentiated between alcohol dependence, alcohol withdrawal, and delirium tremens (with increasing levels in that order) [35]. We also found significantly lower CK activity in females compared to males for patients, as well as for healthy controls (Table 1), which was similarly observed in the animal model [33]. Contrary to the expectation of increased levels, CK was slightly, but not significantly, decreased in patients compared to healthy subjects (Table 1). Thus, CK would not be useful as a biomarker for alcohol dependence, based on our data. However, we observed a positive correlation between CK and S-ASM activity within the group of patients (Table 2), characterized by a stronger contribution from the female group.

### 2.6. S-ASM Activity Is Differentially Associated with Myelosuppression in Patients and Controls

As expected from the strong association of S-ASM activity with hepatotoxicity, as demonstrated by the correlation with liver enzymes, the enzyme activity was also related to myelosuppression in patients (Table 2). In the total cohort of patients (and, particularly, in the male subgroup), higher S-ASM levels correlated with lower leukocyte numbers, which are indicative of the toxic effect of alcohol on hematopoiesis. In contrast, higher S-ASM levels in healthy controls (especially in females) were associated with higher leukocyte numbers, which could reflect inflammatory processes that are known to be related to elevated S-ASM levels [12]. For thrombocytes, higher S-ASM levels were also associated with lower cell counts, and the strongest effects were found in female patients. Here, we did not observe an effect in controls.

### 2.7. S-ASM Activity Is Associated with Alterations in Triglycerides in HDL Cholesterol in Patients

Triglycerides and cholesterol subspecies might interact with the activity of a lipid metabolizing enzyme, such as S-ASM. Levels of triglycerides were slightly increased in female patients and unaltered in males, compared to controls, in contrast to a decrease found in Japanese [36] and in European Americans [37] for low-to-moderate alcohol consumption (Table 1). Triglyceride concentrations correlated negatively with S-ASM activity in our total patient group, as well as in male patients (Table 2), in accordance with previous observations in a smaller study [14].

Both male and female patients’ samples contained significantly higher levels of high-density lipoprotein (HDL) cholesterol (Table 1), in line with published reports for the effect of alcohol intake [36,37,38]. The significant positive correlation between HDL cholesterol levels and S-ASM activity in patients (Table 2), in agreement with previous reports [14], could reflect a causal relationship or an independent influence of alcohol consumption on both parameters. Interestingly, while the concentration of low-density lipoprotein (LDL) cholesterol was also significantly altered in both male and female patients, in line with published data on reduced levels [36,37], unlike for HDL cholesterol, it was not associated with S-ASM activity (Table 1 and Table 2), in accordance with previous results [14]. The analysis of HDL and LDL cholesterol subfractions could provide further insights into these apparently differential relationships with S-ASM activity.

In healthy control subjects, we detected only a relationship between S-ASM and triglycerides in females, with an opposite direction compared to patients, i.e., higher S-ASM activity was associated with higher triglyceride levels (Table 2).

### 2.8. S-ASM Activity Does Not Predict Alcohol-Related Readmission for Patients

Medical records of patients were checked for 24 months after recruitment, to assess alcohol-related readmissions. However, S-ASM activity did not differ between patients with at least one alcohol-related readmission (*n* = 122), and those without a readmission (*n* = 78; Mann–Whitney *U* test, *U* = 4451, *p* = 0.442). Moreover, the S-ASM activity did not predict the days until the first readmission (Rho = −0.042, *p* = 0.555), nor did it predict the number of readmissions (Rho = 0.025, *p* = 0.723). This was also true when male and female patients were analyzed separately. On the other hand, one of the strongest known predictors—the number of previous withdrawal treatments—also strongly predicted alcohol-related readmission in this cohort (Mann–Whitney *U* test, *U* = 1368, *p* = 2.0 × 10^−5^), as well as days until first readmission (Rho = −0.345, *p* < 1.8 × 10^−5^) and the number of readmissions (Rho = 0.400, *p* < 5.3 × 10^−7^).

## 3. Discussion

We have confirmed both the previously described increased activity of S-ASM in alcohol-dependent patients, and the decrease in S-ASM activity during detoxification treatment in a large and sex-balanced cohort [14,15]. However, in a smaller previous study consisting predominantly of males, S-ASM activity in patients was 3-fold higher compared to healthy controls, and it declined in every single individual over 7–10 days of withdrawal treatment by 52% of the initial value, on average [14]. In another small mixed gender study without controls, S-ASM activity fell by 15%–20% for females to 22%–29% for males during 2–7 days of treatment. A possible explanation for the smaller effects, in this study, could be our time window for inclusion during early abstinence (i.e., 24 to 72 h after the last consumption of alcohol), which could already be too late to detect the high initial drop in ASM activity.

While the increase in S-ASM activity in alcohol-dependent patients has been replicated, its origin remains elusive. A wide variety of cells have been demonstrated to secrete substantial amounts of this Zn^2+^-dependent enzyme, including human vascular endothelial cells, macrophages, and platelets [12], resulting in detectable levels not only in the blood, but also in cerebrospinal fluid [39]. In mice fed on an atherogenic diet containing saturated fats and cholesterol, an increased macrophage secretion seemed to be responsible for the elevated S-ASM activity [40]. The enzyme might be released into the bloodstream when cells are injured by ethanol, or other components of alcoholic beverages or as a response to systemic changes induced by these factors. ASM is a key regulator of ceramide-dependent signaling pathways, and it can be induced by cellular stress resulting from inflammation or infection [12]. Mechanisms of ASM activation by ethanol could involve post-translational, as well as transcriptional effects [41].

On the other hand, alcohol-dependent patients could carry risk factors for endogenously higher ASM levels. Genetically determined ASM activity is already known to influence the susceptibility for common human diseases, such as allergy [42]. However, because known single nucleotide polymorphisms or variations in the repeat number within the special signal peptide negatively affect ASM activity [12,43,44], it is rather unlikely that a frequent, but so far undetected, variant within the *SMPD1* gene would predispose carriers to developing alcohol dependence, and be the cause of higher S-ASM levels. On the other hand, processes associated with posttranslational modifications, and regulation that modulate ASM trafficking, maturation, or secretion [45], as well as those leading to degradation of the enzyme, could permanently or temporarily be altered in patients. Additionally, gene variants encoding proteins further upstream in the lipid synthesis pathway, such as in *SERINC2*, could alter ASM levels. This gene was identified as a top-ranked risk gene for alcohol dependence [46,47], and the encoded protein incorporates serine into membranes, facilitating the synthesis of phosphatidylserine and sphingolipids [48]. Moreover, *SMPD1* splicing [49] has been reported to influence ASM activity and be altered in major depression [50]. However, it has not yet been analyzed in alcohol dependence.

Remarkably, GGT values of a considerable proportion of healthy controls (17% of males, 7% of females) were above the normal reference range of the analyzing laboratory, with the upper limit of 60 U/L for males and 40 U/L for females. Moreover, there is evidence from data on blood pressure, pulse rate, relative body weight, and serum insulin, that call for an even lower upper limit of 10 U/L, compared to the 28 U/L limit at the time of the investigation [51]. Consumption of small amounts of alcoholic beverages as “social” drinking, that is not yet detected by the CAGE or AUDIT questionnaires, but is probably the main cause of elevated GGT activity which reflects a low level of liver damage, seems to also be associated with higher S-ASM levels in control subjects. However, the influence of chronic low-dose alcohol exposure appears to be different from the impact of acutely high levels, suggested by the different correlations between GGT and S-ASM after subdividing the healthy control sample according to the presence of binge-drinking episodes. These associations of S-ASM activity with liver enzyme activities warrant further investigation.

We detected a significant correlation between S-ASM and each patient’s alcohol levels at admission. A greater effect was observed in the male subsample, but no relationship was found with the predominantly consumed type of alcoholic beverage. It is also noteworthy that there were different correlation strengths for S-ASM and various classical biomarkers of alcohol-dependence. While the association was very strong for liver enzymes and CDT, it was much weaker for the well-accepted indicator, MCV. Similarly, significant correlations between ASM and the indicators of hepatic injury (GGT, ALT, and AST) have also been described for patients with a hepatitis C virus infection where ASM showed a high discriminative power [23]. There could be a common link between ASM and hepatotoxicity that involves endoplasmic reticulum stress and cholesterol loading of mitochondria [52] that are highly abundant in hepatocytes, possibly via mechanisms of transcriptional regulation recently identified for mitochondrial defects in lysosomal storage disorders, like Niemann–Pick disease caused by a genetic defect in the ASM encoding gene (Yambire K.F. et al. preprint under revision).

Withdrawal symptoms, assessed during the follow-up visit using the CIWA-Ar scale, were significantly related to S-ASM levels, and this correlation was stronger in females than in males. On the other hand, the strong and highly significant positive relationship, between the sub-item tremor during detoxification and S-ASM, was most prominent in males. Interestingly, when beta-endorphin levels from the same cohort were analyzed with respect to withdrawal severity, the female subgroup contributed mostly to the correlation of the sub-item score for impaired concentration with higher initial beta-endorphin levels, as well as a stronger decline during withdrawal [53]. These sex-specific effects once more emphasize the importance of separate analyses for males and females in the field of alcohol addiction.

Although there is a need for very cautious interpretation due to the clearly different mechanisms at work, there is an intriguing association between tremor and ASM in a very different disorder—Parkinson’s disease—with tremor being one of the primary early symptoms. Following the initial identification of the rare p.L302P mutation in *SMPD1* as a strong risk factor for Parkinson’s disease in Ashkenazi Jews [54], two additional *SMPD1* founder mutations were identified in this population [55]. A pathogenic mechanism for Parkinson’s disease has been hypothesized that involves alterations of the autophagy–lysosome pathway based on additional genetic factors encoding lysosomal enzymes [56].

Biomarkers for alcohol consumption, that are more reliable than self-reports and physiological assessments, are essential not only for diagnosis and treatment of alcohol-related disorders, but also for epidemiological studies of the health effects of alcohol itself, or of other exposure events with alcohol as a cofactor. No gold standard is yet available, and commonly applied biochemical markers are far from ideal with respect to their discriminatory power, as indicated by AUC values ranging from 0.21 to 0.67 [29]. While new approaches, including protein markers [29], DNA methylation patterns [57], aldehyde-induced DNA, and protein adducts [58], and even neuroimaging [59,60] enhance the detection of heavy drinkers, there is still room for improvement, for example, by utilizing novel biomarkers or by developing of a composite score for excessive alcohol use screening [61]. In our study population, S-ASM activity alone did not perform better than any of the analyzed markers (Table 2, AUC = 0.77 compared to AUC ≥ 0.80 for conventional biomarkers in our cohort), and it also did not appear to be beneficial when this information was added to a combined model. However, S-ASM elevation might respond to a different threshold of alcohol consumption and/or span a different time window than CDT, MCV, or GGT. So far, only the gradual decrease during the short period of withdrawal treatment of up to 10 days has been described, characterized by a reduction ranging from 6% to 52% of the initial value, in this and previous studies [14,15]. The rate of decline would be relevant in monitoring for relapse. The response time of S-ASM to different amounts and types of alcohol intake has not been investigated yet.

There was no indication that S-ASM levels are suitable to predict 24-month alcohol relapse, although the collected data appeared valid, as suggested by the observed and known strong predictive value of the number of previous withdrawal treatments. Further predictive factors have also been identified in this NOAH cohort. For example, a single nucleotide variant in *OPRM1*, which encodes the mu opioid receptor binding the endogenous ligand beta-endorphin, was associated with an increased risk of more and earlier alcohol-related hospital readmissions [53]. Moreover, a higher body mass index in male patients and higher craving scores [62], as well as clinical Cloninger and Lesch typology classifications [63] are suggested as easily accessible risk factors and promising tools. The failure of S-ASM to serve as a useful predictor of alcohol relapse, however, does not imply that other components and enzymes of the sphingolipid pathway could not serve as biomarkers. Analysis of their activities or of the serum sphingolipid profile has, thus, some potential, given the relationships of S-ASM with the alcohol biomarkers observed in this study.

Although offering promising results, our study has some limitations beyond the limited sample size. The group of healthy controls was not abstinent and might not be representative because they were recruited from a largely academic environment. They also differ from patients in parameters such as BMI, smoking, and certainly nutrition, including supply with vitamins, which could have an additional influence on the observed effects. Many patients had to be excluded during the screening process and, thus, the generalization of the patient data might also be limited. The frequency of relapse is certainly underestimated, and could contribute to the observed lack of an effect of ASM because we relied on medical records for readmission to the two study centers. We, therefore, may have missed patients treated at other centers, or who did not seek out medical advice at all. Our data need to be interpreted with caution because they do not reflect causal relationships, but are instead associational. Future studies should investigate the potential causation of these findings. Some aspects warrant verification in cell culture or animal models. Due to the explorative nature of the study, we have not corrected the *p*-values for multiple testing. As such, some strong and nominally highly significant associations would survive strict corrections, but still require independent verification in larger cohorts of mixed sexes, if possible.

## 4. Materials and Methods

### 4.1. Cohort Characteristics

This investigation was part of the bicentric, cross-sectional, and prospective Neurobiology of Alcoholism (NOAH) study [32]. In 2013 and 2014, a sex-balanced cohort of 200 alcohol-dependent in-patients seeking withdrawal treatment was recruited at the Universitätsklinikum Erlangen Department of Psychiatry and Psychotherapy, and the Klinikum am Europakanal Clinic for Psychiatry, Addiction, Psychotherapy, and Psychosomatic Medicine in Erlangen, Germany. Each patient was diagnosed with an alcohol-use disorder according to the fifth edition of the Diagnostic and Statistical Manual of Mental Disorders (DSM-5, American Psychiatric Association, 2013) and alcohol dependence, according to the tenth revision of the International Classification of Diseases (ICD-10, World Health Organization, 1992). In addition, we recruited 240 healthy control subjects who underwent a multi-step screening procedure to exclude severe somatic and psychiatric morbidity (with the exclusion of nicotine dependence) (for details see [32]). The study was approved by the Ethics Committee of the Medical Faculty of the Friedrich-Alexander University Erlangen-Nürnberg (NOAH study ID 81_12 B, 19 April 2012). All participants provided written informed consent.

The study inclusion with the first blood draw took place during early abstinence (24 to 72 h of abstinence). Afterwards, 81.5% of the patients participated at a direct follow-up at median 5 days later (interquartile range (IQR) 3–6), which included a second blood draw. Whole blood, behavioral scores, and other parameters [32] were collected at the time of recruitment. The German version of the Clinical Institute Withdrawal Assessment for Alcohol revised (CIWA-Ar) scale was used to measure alcohol withdrawal severity in patients at the follow-up [64]. The patients’ records were followed for 24 months post-inclusion, to investigate alcohol-related readmissions. In the healthy controls, potential problems of alcohol consumption were assessed by the 4-item CAGE questionnaire [65], and the 10-item Alcohol Use Disorders Identification Test (AUDIT) [66]. The study sample characteristics are provided in Table 1.

### 4.2. Blood Analysis

Blood samples were collected in the morning for all individuals to minimize circadian effects on hormone levels. Serum vials were centrifuged (10 min and 2000× *g* at room temperature), and serum was aliquoted and placed into storage at −80 °C for later S-ASM activity assays. Glutamic oxaloacetic transaminase (GOT), glutamic-pyruvic transaminase (GPT), gamma-glutamyl transferase (GGT), and creatine kinase (CK) activities, as well as leukocyte and thrombocyte counts, triglycerides, total, HDL and LDL cholesterol, were quantified at the Central Laboratory of the Universitätsklinikum Erlangen, Germany (DIN EN ISO 15189 accredited), from separately collected serum and EDTA vials. Except for one patient who underwent a direct measurement, blood alcohol concentrations were estimated from breath alcohol content that was determined and documented upon admission to the hospital.

### 4.3. Determination of S-ASM Activity

The activity of S-ASM was quantified using the fluorescent substrate BODIPY-FL-C12-SM (*N*-(4,4-difluoro-5,7-dimethyl-4-bora-3a,4a-diaza-s-indacene-3-dodecanoyl)sphingosyl phosphocholine, D-7711, Thermo Fisher Scientific, Waltham, MA, USA), as described previously [67]. Briefly, the reaction was performed in 96-well polystyrene plates with 58 pmol sphingomyelin in a reaction buffer totaling 50 µL in volume, in the following composition: 200 mM sodium acetate buffer (pH 5.0), 500 mM NaCl, 0.2% IGEPAL® CA-630 (NP 40), and 500 µM ZnCl_2_. The reaction was initiated by the addition of 6 µL of a 1:10 dilution of serum in physiological 154 mM NaCl solution. After incubation at 37 °C for 24 h, reactions were stopped by freezing at −20 °C, and stored until further processing. For direct chromatography, 1.5 µL of the reaction was spotted directly without further purification on silica gel 60 thin layer chromatography plates (ALUGRAM SIL G, 818232, Macherey-Nagel, Düren, Germany). Product and uncleaved substrate were separated using ethyl acetate with 1% (*v*/*v*) acetic acid as a solvent. Spot intensities were detected on a Typhoon Trio scanner, and quantified using the ImageQuant software (GE Healthcare Life Sciences, Buckinghamshire, UK). All enzyme activity assays were carried out with four replicate dilutions of each sample, and using the same lot of reagents and consumables and performed by a single operator.

### 4.4. Statistics

Data were analyzed using IBM SPSS Statistics Version 21 for Windows (SPSS Inc., Chicago, IL, USA) and GraphPad Prism 7.00 (GraphPad Software Inc., San Diego, CA, USA). Continuous data are presented as the median and IQR in tables as calculated by the custom tables function of SPSS. In the case of missing data points (percentage indicated in Table 1), study subjects were excluded from the specific analyses. Spearman correlations were employed to evaluate associations between two continuous variables. Differences between groups were tested using the Mann–Whitney *U* test because the values were not normally distributed according to the Kolmogorov–Smirnov test. Differences between alcohol-dependent patients’ enzyme activities at different time points were tested using the Wilcoxon signed-rank test. *p*-Values less than 0.05 for two-sided tests were considered statistically significant. Receiver operating characteristic curve analysis was used to estimate S-ASM activity and alcohol biomarkers required to separate alcohol-dependent patients from healthy controls (including AUC, Youden cut-point, and related sensitivity and specificity). Female and male patients were analyzed separately because of the well-established sex differences in alcohol dependence [68,69] and the highly significant differences in many of the investigated parameters between males and females (Table 1).

## 5. Conclusions

We replicated our previous observation of increased S-ASM activity in male and female alcohol-dependent patients by examining a large cohort. We characterized previously unreported associations among S-ASM, alcohol levels, and alcohol withdrawal, as well as biomarkers of alcohol dependence. These associations were not only observed in patients but, to some extent, in healthy controls as well. While most effects were similarly present in male and female subgroups, some differences emphasized the necessity to sex-specificity when analyzing the data. Further research should investigate whether there is a causal relationship, or whether these parameters are part of a common pathway, in order to gain insights into the underlying mechanisms and to develop clinical applications.

Increased lysosomal and S-ASM activity in alcohol-dependent patients could lead to elevated ceramide concentrations in the brain. Ceramide is assumed to act as a negative regulator of neurogenesis, neuronal maturation, and survival [70]. Thus, a reduction by pharmacological means (most common antidepressants act as functional inhibitors of ASM [71,72]) to normalize levels could improve these pathologies. A less direct effect could be achieved by nutritional changes, such as supplementation with docosahexaenoic acid during detoxification, to potentially protect against dependence-related neuroinjury [73,74]. A more comprehensive molecular understanding of the alterations of sphingolipid metabolism in the context of alcohol dependence would provide a basis for the rational development of new drugs and treatments.

## Figures and Tables

**Table 1:** Demographic and laboratory data for male and female alcohol-dependent patients and healthy control subjects.

Parameter	Alcohol-Dependent Patients		Healthy Control Subjects		*p* Patients vs. Controls		*p* ♂ vs. ♀	
	♂	♀	♂	♀	♂	♀	Patients	Controls
*n*	113	87	133	107	-	-	-	-
Age (years)	48 (40–53)	48 (42–55)	48 (38–56)	49 (39–55)	0.794	0.772	0.308	0.762
BMI (kg/m^2^) ^b^	24.9 (22.1–27.8)	24.4 (22.1–29.3)	27.7 (24.9–29.5)	25.0 (21.7–28.6)	**<0.001**	0.961	0.963	**<0.001**
Active smokers (%) ^b^	77.9	76.9	21.8	18.7	**<0.001**	**<0.001**	**<0.001**	**<0.001**
Age at onset of alcohol dependence (years) ^c^	30 (24–39)	35 (28–42)	-	-	-	-	**0.015**	-
Previous withdrawal treatments (*n*) ^c^	6 (2–12)	5 (2–11)	-	-	-	-	0.892	-
Alcohol concentration at admission (‰) ^b^	1.7 (0.5–2.4)	1.2 (0.1–1.8)	-	-	-	-	**0.020**	-
CIWA-Ar total score ^c^	18 (15–23)	16 (14–21)	-	-	-	-	0.163	-
CAGE score	-	-	0 (0–1)	0 (0–0)	-	-	-	**0.024**
AUDIT score ^b^	-	-	4 (3–6)	3 (2–4)	-	-	-	**<0.001**
Days until first alcohol-related readmission	285 (57–730)	625 (90–730)	-	-	-	-	**0.047**	-
Number of alcohol-related readmissions	2 (0–4)	1 (0–3)	-	-	-	-	**0.021**	-
S-ASM activity during recruitment (fmol/h/µL serum)	224 (168–318)	202 (157–256)	150 (112–194)	134 (97–181)	**<0.001**	**<0.001**	**0.049**	0.100
S-ASM activity during follow-up (fmol/h/µL serum) ^b^	214 (142–299)	183 (159–251)	-	-	-	-	0.175	-
GGT (U/L)	109 (50–275)	57 (34–230)	28 (21–41)	17 (14–25)	**<0.001**	**<0.001**	**0.022**	**<0.001**
ALT (U/L)	48 (28–84)	28 (20–50)	30 (23–39)	18 (14–24)	**<0.001**	**<0.001**	**<0.001**	**<0.001**
AST (U/L)	51 (36–91)	37 (28–67)	29 (25–34)	23 (20–26)	**<0.001**	**<0.001**	**0.002**	**<0.001**
CDT (nephelometry, %) ^a^	2.8 (1.9–4.0)	1.9 (1.6–2.5)	1.5 (1.3–1.7)	1.5 (1.3–1.6)	**<0.001**	**<0.001**	**<0.001**	0.486
MCV (fl) ^a^	93 (90–96)	95 (91–97)	88 (85–91)	88 (85–91)	**<0.001**	**<0.001**	0.166	0.734
Homocysteine (µmol)	15 (12–23)	15 (11–21)	12 (10–14)	10 (9–12)	**<0.001**	**<0.001**	0.161	**<0.001**
CK (U/L)	132 (81–220)	89 (72–141)	151 (112–213)	92 (75–110)	0.058	0.422	**0.006**	**<0.001**
Leukocytes (per nL) ^a^	7.2 (5.5–8.4)	6.9 (5.2–8.6)	5.8 (5.0–7.2)	5.8 (4.7–6.7)	**<0.001**	**<0.001**	0.341	0.173
Thrombocytes (per nL) ^a^	198 (145–254)	218 (181–266)	230 (196–257)	251 (214–287)	**<0.001**	**<0.001**	**0.021**	**<0.001**
Triglycerides (mg/dL)	165 (96–236)	135 (95–221)	135 (98–192)	117 (80–165)	0.244	**0.012**	0.479	**0.008**
Total cholesterol (mg/dL)	214 (185–252)	216 (180–255)	210 (189–239)	223 (191–252)	0.833	0.530	0.926	0.106
High-density lipoprotein cholesterol (mg/dL)	61 (51–80)	68 (54–85)	48 (43–57)	62 (51–74)	**<0.001**	**0.015**	0.123	**<0.001**
Low-density lipoprotein cholesterol (mg/dL) ^a^	130 (96–160)	122 (98–157)	143 (125–171)	146 (122–166)	**<0.001**	**<0.001**	0.750	0.676

The table shows medians with interquartile range or percentages, and *p*-values from Mann–Whitney *U* tests and χ^2^ tests comparing sex-specifically early-abstinent alcohol-dependent patients with healthy control subjects, and comparing group-specific male to female individuals. The subgroup at follow-up (94 male and 69 female patients willing and able to participate 5 days (3–6) after the initial recruitment) is representative for the total cohort of alcohol-dependent patients, since there are no significant differences in sociodemographic characteristics [32] or regarding S-ASM activity (all sex-specific *p* < 0.1). Data on alcohol-related readmissions were extracted from medical records for the 24-month period after study recruitment. For patients without readmission, this value was set to 730 days. Missing values: a ≤ 1%, b ≤ 10%, c ≤ 30%. *p* < 0.05 in bold. BMI: body mass index, CIWA-Ar score: German version of the Clinical Institute Withdrawal Assessment for Alcohol revised, CAGE: acronym for 4-item questionnaire indicating potential problems with alcohol abuse, AUDIT: Alcohol Use Disorders Identification Test, S-ASM: secretory acid sphingomyelinase, GGT: gamma-glutamyl transferase, ALT: alanine aminotransferase (glutamic-pyruvic transaminase, GPT), AST: aspartate aminotransferase (glutamic-oxaloacetic transaminase, GOT), CDT: carbohydrate-deficient transferrin, MCV: mean corpuscular volume, CK: creatine kinase.

**Table 2:** Total and sex-specific correlations of peripheral S-ASM activity with blood parameters altered in alcohol-dependent patients.

Parameter	Patients		Controls		Patients				Controls			
					♂		♀		♂		♀	
	Rho	*p*	Rho	*p*	Rho	*p*	Rho	*p*	Rho	*p*	Rho	*p*
GGT	0.373	**5.6 × 10^−8^**	0.254	**7.0 × 10^−5^**	0.327	**4.4 × 10^−4^**	0.378	**3.1 × 10^−4^**	0.217	**0.012**	0.307	**0.001**
ALT	0.373	**5.4 × 10^−8^**	0.170	**0.008**	0.327	**4.0 × 10^−4^**	0.368	**4.6 × 10^−4^**	0.124	0.154	0.177	0.068
AST	0.499	**5.4 × 10^−14^**	0.122	0.060	0.465	**2.2 × 10^−7^**	0.524	**1.9 × 10^−7^**	0.092	0.291	0.093	0.340
CDT	0.378	**3.4 × 10^−8^**	−0.062	0.341	0.311	**7.9 × 10^−4^**	0.400	**1.2 × 10^−4^**	0.030	0.732	−0.162	0.095
MCV	0.172	**0.016**	0.139	**0.032**	0.154	0.104	0.248	**0.022**	0.170	**0.050**	0.085	0.388
Homocysteine	0.304	**1.1 × 10^−5^**	0.142	**0.028**	0.369	**5.7 × 10^−5^**	0.201	0.062	0.123	0.158	0.092	0.347
CK	0.218	**0.002**	0.008	0.902	0.167	0.076	0.228	**0.033**	0.015	0.868	−0.114	0.243
Leukocytes	−0.152	**0.033**	0.145	**0.025**	−0.248	**0.008**	−0.059	0.591	0.093	0.287	0.209	**0.032**
Thrombocytes	−0.292	**3.0 × 10^−5^**	0.015	0.812	−0.229	**0.015**	−0.365	**6.0 × 10^−4^**	0.030	0.732	0.070	0.475
Triglycerides	−0.214	**0.002**	0.126	0.052	−0.233	**0.013**	−0.188	0.082	−0.004	0.965	0.252	**0.009**
Cholesterol	0.055	0.435	0.027	0.679	0.097	0.308	0.022	0.838	0.034	0.698	0.058	0.551
HDL cholesterol	0.229	**0.001**	0.004	0.954	0.272	**0.004**	0.215	**0.045**	0.139	0.112	−0.086	0.379
LDL cholesterol	−0.050	0.485	0.017	0.797	−0.035	0.715	−0.072	0.509	−0.034	0.700	0.081	0.405

Rho and *p*-values from Spearman correlations. *p* < 0.05 in bold. S-ASM: secretory acid sphingomyelinase, GGT: gamma-glutamyl transferase, ALT: alanine aminotransferase (glutamic-pyruvic transaminase, GPT), AST: aspartate aminotransferase (glutamic-oxaloacetic transaminase, GOT), CDT: carbohydrate-deficient transferrin, MCV: mean corpuscular volume, CK: creatine kinase, HDL high-density lipoprotein, LDL low-density lipoprotein.

**Table 3:** ROC analysis for S-ASM, common alcohol biomarkers, and alcohol-dependent routine blood parameters.

	Total Cohort				♂				♀			
	AUC	Y	Sens	Spec	AUC	Y	Sens	Spec	AUC	Y	Sens	Spec
S-ASM (fmol/h/µL)	0.771	151	0.850	0.554	0.787	224	0.504	0.910	0.753	151	0.816	0.607
CDT (%)	0.868	1.72	0.760	0.849	0.890	1.77	0.796	0.841	0.848	1.71	0.690	0.888
GGT (U/L)	0.853	39.5	0.740	0.817	0.843	44.0	0.779	0.774	0.886	33.5	0.770	0.869
MCV (fl)	0.799	90.1	0.787	0.695	0.778	90.0	0.777	0.684	0.824	91.8	0.729	0.830
ALT (U/L)	0.687	47.5	0.410	0.904	0.687	47.5	0.522	0.857	0.725	22.5	0.621	0.738
AST (U/L)	0.806	33.5	0.700	0.821	0.816	35.5	0.752	0.812	0.837	26.5	0.782	0.776
Hcy (µmol)	0.758	13.2	0.650	0.750	0.742	13.0	0.717	0.669	0.783	12.2	0.667	0.785

ROC: receiver operating characteristic, AUC: area under the curve, Y: Youden cut-point, Sens: sensitivity, Spec: specificity, S-ASM: secretory acid sphingomyelinase, CDT: carbohydrate-deficient transferrin, GGT: gamma-glutamyl transferase, MCV: mean corpuscular volume, ALT: alanine aminotransferase (glutamic-pyruvic transaminase, GPT), AST: aspartate aminotransferase (glutamic-oxaloacetic transaminase, GOT).

## Supplementary Materials

The following are available online at http://www.mdpi.com/1422-0067/19/12/4028/s1, Table S1: Total and sex-specific correlations of peripheral S-ASM activity with sub-items of the Clinical Institute Withdrawal Assessment for Alcohol revised scale (CIWA-Ar) in alcohol-dependent patients, Table S2: Activity of serum acid sphingomyelinase (S-ASM) in control subjects subdivided according to the presence or absence of at least one binge-drinking episode within the past 24 months. Figure S1: Sex-specific activity of the secretory acid sphingomyelinase (S-ASM) in alcohol-dependent male and female patients subdivided according to their predominantly consumed type of alcoholic beverage during early abstinence. Boxplots show individual data, and the median and interquartile range. The numbers of male and female individuals is provided below the x-axis.

## Abbreviations

ALT	alanine aminotransferase (glutamic-pyruvic transaminase, GPT)
AST	aspartate aminotransferase (glutamic-oxaloacetic transaminase, GOT)
AUC	area under the curve
AUDIT	Alcohol Use Disorders Identification Test
BMI	body mass index
CAGE	acronym for 4-item questionnaire indicating potential problems with alcohol abuse
CDT	carbohydrate-deficient transferrin
CIWA-Ar score	Clinical Institute Withdrawal Assessment for Alcohol revised score
CK	creatine kinase
GGT	gamma-glutamyl transferase
Hcy	homocysteine
HDL	high-density lipoprotein
IQR	interquartile range
LDL	low-density lipoprotein
MCV	mean corpuscular volume
S-ASM	secretory acid sphingomyelinase
*SMPD1*	sphingomyelin phosphodiesterase 1 gene encoding ASM
