# Supplementary material for: Peripheral Acid Sphingomyelinase Activity Is Associated with Biomarkers and Phenotypes of Alcohol Use and Dependence in Patients and Healthy Controls
Source: Int J Mol Sci. 2018 Dec 13;19(12):4028. doi: 10.3390/ijms19124028 (PMC6320816; doi:10.3390/ijms19124028)
Supplement: Supplementary file 1 [file ijms-19-04028-s001.pdf]

Article

Peripheral acid sphingomyelinase activity is associated with biomarkers and phenotypes of alcohol use and dependence in patients and controls

Christiane Mühle[1*] (ORCID 0000-0001-7517-9154), Christian Weinland[1] (ORCID 0000-0002-0007-4902), Erich Gulbins[2], Bernd Lenz[1,†] (ORCID 0000-0001-6086-0924), Johannes Kornhuber[1,†] (ORCID 0000-0002-8096-3987)

Table S1. Total and sex-specific correlations of peripheral S-ASM activity with sub-items of the Clinical Institute Withdrawal Assessment for Alcohol revised scale (CIWA-Ar) in alcohol-dependent patients.

	total group (n=146)		♂ (n=83)		♀ (n=63)	
CIWA-Ar sub-item	Rho	p	Rho	p	Rho	p
nausea/vomiting	0.232	**0.005**	0.261	**0.017**	0.209	0.100
tremor	0.351	**1.4×10^{-5}**	0.395	**2.4×10^{-4}**	0.250	**0.048**
hyperhidrosis	0.195	**0.018**	0.146	0.186	0.225	0.076
tactile disturbances	0.005	0.953	-0.034	0.763	0.006	0.965
auditory disturbances	-0.006	0.947	-0.052	0.638	0.058	0.653
visual disturbances	-0.032	0.701	-0.106	0.340	0.068	0.599
orientation	0.088	0.290	0.110	0.320	-0.023	0.859
concentration	0.226	**0.006**	0.185	0.095	0.283	**0.024**
nervousness/anxiety	0.077	0.357	0.019	0.863	0.151	0.236
headache	-0.078	0.348	-0.126	0.258	0.038	0.770
CIWA-Ar total	0.242	**0.003**	0.194	0.079	0.267	**0.034**

Table S2. Activity of serum acid sphingomyelinase (S-ASM) in control subjects subdivided according to the presence or absence of at least one binge-drinking episode within the past 24 months.

	Binge drinkers		Non-binge drinkers	
	♂	♀	♂	♀
n	29	7	104	100
Age (years)	41 (34 - 52)	47 (28 - 54)	48 (39 - 57)	49 (40 - 55)
S-ASM activity (fmol/h/µl serum)	139 (97 - 177)	128 (110 - 219)	156 (121 - 197)	135 (97 - 181)

Medians with interquartile range.

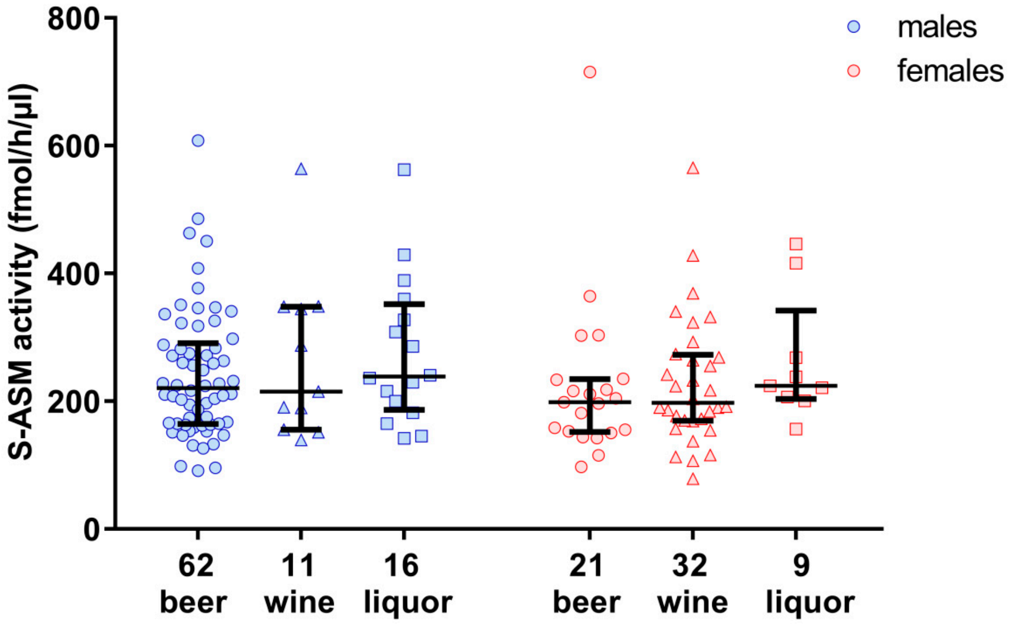

Supplemental Figure S1. Sex-specific activity of the secretory acid sphingomyelinase (S-ASM) in alcohol-dependent male and female patients subdivided according to their predominantly consumed type of alcoholic beverage during early abstinence. Boxplots show individual data, and the median and interquartile range. The numbers of male and female individuals is provided below the x-axis.
